# The Application of Circulating Tumour DNA (ctDNA) in the Diagnosis, Prognosis, and Treatment Monitoring of Gynaecological and Breast Cancers (Review)

**DOI:** 10.3390/diagnostics15101289

**Published:** 2025-05-21

**Authors:** Aleksandra Englisz, Marta Smycz-Kubańska, Patrycja Królewska-Daszczyńska, Magdalena Błaut, Agnieszka Duszyc, Aleksandra Mielczarek-Palacz

**Affiliations:** 1 The Doctoral School, Medical University of Silesia, 40-055 Katowice, Poland; d201166@365.sum.edu.pl; 2 Department of Immunology and Serology, Faculty of Pharmaceutical Sciences in Sosnowiec, Medical University of Silesia, 40-055 Katowice, Poland; mkubanska@sum.edu.pl (M.S.-K.); pdaszczynska@sum.edu.pl (P.K.-D.); mblaut@wp.pl (M.B.); agnieszka.duszyc@sum.edu.pl (A.D.)

**Keywords:** gynaecological cancers, breast cancer, biomarkers, ctDNA

## Abstract

Gynaecological cancers, including endometrial, ovarian, and cervical cancers as well as breast cancer, despite numerous studies, still constitute a challenge for modern oncology. For this reason, research aimed at the application of modern diagnostic methods that are useful in early detection, prognosis, and treatment monitoring deserves special attention, Great hopes are currently being placed on the use of liquid biopsy (LB), which examines various tumour components, including cell-free RNA (cfRNA), circulating tumour cells (CTCs), circulating tumour DNA (ctDNA), exosomes, and tumour-educated platelets (TEPs). LB has shown promise as a minimally invasive means of early diagnosis of cancers, detection of recurrence, prediction of therapy response, treatment monitoring, and drug selection. The integration of this test into clinical practice in modern oncology is challenging, but offers many benefits, including reducing the risks associated with invasive procedures, improving diagnostic and therapeutic efficacy, and improving the quality of life of oncology patients. The aim of this review is to present recent reports on the use of ctDNA in diagnosing, predicting the outcome of, and monitoring the treatment of gynaecological and breast cancers.

## 1. Introduction

**Figure 1:**
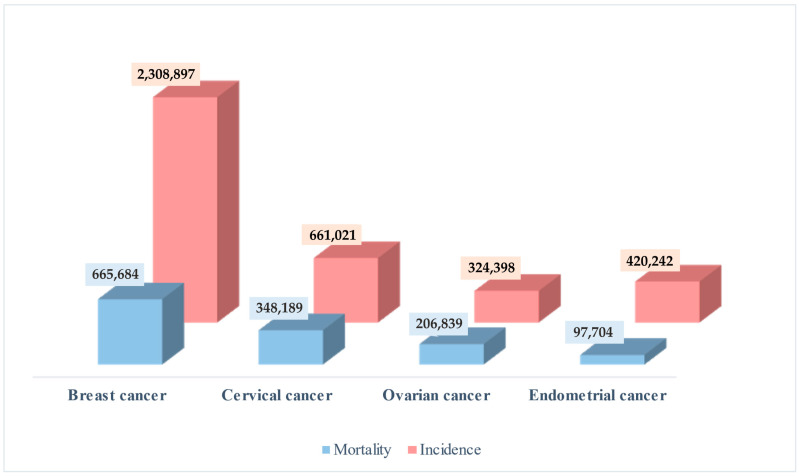
Global gynaecological cancers and breast cancer morbidity and mortality in 2022.

Laboratory methods for determining the concentration of biomarkers specific for individual cancers, as well as imaging methods and examination of tissues collected by biopsy, are used in the diagnosis and treatment monitoring of cancer. The primary goal is to identify biomarkers that can be used as an effective screening test, particularly in the early stages of the disease, help develop new diagnostic and therapeutic strategies, and, importantly, can be determined using minimally invasive methods. Currently, researchers are paying attention to the liquid biopsy (LB) method, which allows for serial sampling to monitor the progression of the disease in real time and has the potential to improve oncological diagnostics. This review summarises c reports on the potential use of ctDNA as a biomarker of carcinogenesis and progression of gynaecological and breast cancers, which still constitute a challenge for modern oncology. Figure 1 presents the incidence and mortality rates of gynaecological and breast cancers on a global scale in 2022, as documented in the Global Cancer Statistics, which represent estimates of incidence and mortality rates for 36 cancers in 185 countries worldwide [1].

### 1.1. Liquid Biopsy

LB measures various tumour components, including ctDNA, cfRNA, CTCs, TEPs, and exosomes [2,3]. The most commonly studied biofluid samples include blood, cerebrospinal fluid (CSF), urine, saliva, amniotic fluid, and ascitic fluid [4]. The isolation of ctDNA is also possible from a variety of biological samples, including lymphatic and peritoneal fluids, prostatic fluid, biliary fluid, gastric juice, bone marrow aspirates, peritoneal lavage, sputum, breast milk, and stool samples [5]. LB is a less expensive method than tissue biopsies and imaging methods. Compared to histological examinations, LB is a relatively rapid method and assessment of mutations is possible across tumour tissues that are histologically heterogeneous [4]. In addition, it is a minimally invasive, highly sensitive method that is cheaper in terms of sample isolation costs compared to tissue biopsies. Liquid biopsies can be taken serially to monitor changes with treatment providing an easier means of monitoring treatment response than tissue biopsies. In addition, they have the potential for the detection of material from multiple metastatic sites [6]. The researchers also point out the limitations of this method. The numbers of CTCs, ctDNA, RNA, progenitor and mature endothelial cells, and tumour-derived platelets are usually much lower than the non-cancer DNA fragments in the blood, making them very difficult to detect. Furthermore, standardised methods or protocols for plasma isolation and interpretation are also lacking at present [7]. In clinical practice, LB can be used to diagnose cancers at an early stage, detect recurrence, predict therapy response, monitor treatment, and for drug selection. In addition, it can be used to identify markers that allow patients to be stratified, resulting in personalised therapy [8].

### 1.2. ctDNA

Cell-free DNA (cfDNA) is released via the apoptosis and necrosis of tumour cells [3,9], but the precise mechanism is not fully understood. Elevated cfDNA levels have been observed following exhaustive physical activity, in inflammation, and sepsis [3,9], as a result of surgery, radiotherapy, trauma, or during pregnancy [2]. cfDNA levels are elevated in cancer patients compared to healthy individuals [9]. CTCs, which are cells that have detached from the primary tumour, enter the bloodstream where they release ctDNA. This is a subset of cfDNA, constituting only 1% or even <0.01% of cfDNA [10]. The structural sequence of and epigenetic variations in ctDNA fragments are identical to those of tumour cells [11], and tumour-specific molecular alterations such as mutations, translocations, loss of heterozygosity, copy number alterations, and methylations are carried by ctDNA [12]. The characteristic alterations in the genome may include single-nucleotide variants (SNVs), copy number variations (CNVs), promoter methylation, chromosomal structural rearrangements and alterations in sites relevant to transcription, splicing, RNA maturation, and translation efficiency [5]. Genetic mutations are an important component of ctDNA detection as they have the potential to activate oncogenes and disrupt the balance between oncogenes and tumour suppressor genes, which in turn initiate tumorigenesis. As the abundance of ctDNA is very low, the use of highly sensitive techniques for the detection of tumour mutations is crucial. The methods used in ctDNA analysis include next-generation sequencing (NGS), quantitative polymerase chain reaction (qPCR), digital PCR (dPCR), and droplet digital PCR (ddPCR) [10]. The characteristics of cfDNA and ctDNA molecules are shown in Table 1 [10,11,13,14,15,16].

The usefulness of ctDNA in diagnosing, treating, and predicting the prognosis of cancers has been analysed, showing that it is more sensitive than previously used biomarkers [13]. ctDNA has been analysed in different cancers, including ovarian cancer [11], breast cancer [17,18], lung cancer [19], colon cancer [20], prostate cancer [21], urothelial cancer [22], brain lymphomas [23], and cervical cancer [24]. Using dPCR technology, Bettegowda et al. [25] evaluated the ability of ctDNA to detect tumours in patients with various cancer types (*n* = 640). They demonstrated that ctDNA was detectable in over 75% of patients with advanced pancreatic, bladder, ovarian, breast, colorectal, gastroesophageal, hepatocellular, melanoma, and head and neck cancers. However, ctDNA was detectable in less than 50% of patients with primary brain, renal, prostate, and thyroid cancers. The authors also found that in a group of 206 patients with metastatic colorectal cancers, the sensitivity of ctDNA for detection of clinically relevant mutations in the *KRAS* gene was 87.2% while specificity was 99.2% [25]. It has been shown that ctDNA is a more sensitive indicator of colorectal cancer than serum carcinoembryonic antigen (CEA) [5]. In view of the short half-life of ctDNA compared to CA-125 (estimated half-life range is from 9 to 44 days), its levels in ovarian cancer have therefore been found to be more specific and accurate, providing a real-time picture of tumour burden. This makes ctDNA a more reliable biomarker of disease status and treatment response [9]. Depending on the analytical techniques used for ctDNA detection, the sensitivities and specificities were, respectively, 30–85% and 90–100%, compared to tissue genotyping [26]. The sensitivity (SN) and specificity (SP) of ctDNA assays are shown in Table 2 [27,28,29,30].

ctDNA analyses have been shown to be a promising parameter for screening, early diagnosis, monitoring disease progression, prognosis evaluation, and monitoring response to treatment [31]. This review presents the latest knowledge on the application of ctDNA in diagnosis, prognosis, and treatment monitoring of gynaecological and breast cancers.

## 2. Use of ctDNA as a Biomarker in Gynaecological Cancers and Cancers of the Breast

### 2.1. Ovarian Cancer (OC)

Ovarian cancer is the fifth most common cause of cancer-related deaths among women [32]. According to the 2020 WHO classification, there are at least five main histotypes of ovarian carcinomas: high-grade serous ovarian cancer (HGSC), which accounts for about 70% of ovarian carcinomas; clear cell ovarian carcinoma (CCC)—6–10%; endometrioid ovarian carcinoma (ENOC)—10%; mucinous carcinoma (MC)—3–4%; and low-grade serous carcinoma (LGSC)—5% [32,33]. However, each of these histotypes has a unique genomic profile, resulting in specific clinical behaviour [31]. HGSC is the most deadly histotype, with a five-year survival rate of less than 50% [34]. Based on clinical, pathological, and molecular studies, Shih and Kurman [35] proposed a model that classifies ovarian tumours as either type I or type II. Type I tumours are low-grade neoplasms that are usually diagnosed in the early stages and are typically confined to the ovaries (stage I). They have a favourable prognosis and account for 10% of deaths related to ovarian cancer. Type II tumours are aggressive and high-grade neoplasms, characterised by a rapid rate of growth, have a poor prognosis, and are responsible for 90% of deaths related to ovarian cancer [35,36,37].

Ovarian cancer poses a significant challenge for gynaecological oncology. In the early stages, the course is asymptomatic, and the non-specific symptoms contribute to the late diagnosis, at stages III and IV of the disease. The main risk factors include age at diagnosis [38], genetic predisposition, BRCA1 and BRCA2 mutations, and family history of the cancer [39]. The long-term use of oestrogen therapy for postmenopausal women is considered to be related to an increased risk of endometrioid and serous ovarian cancers [40], while other studies have not found such a relationship [41,42]. The increased risk of ovarian cancer has been reported in nulliparous women, smokers, and women who consume more fat and animal meat [43,44,45]. There are other, more controversial factors to consider, such as obesity, talc powder, infertility, fertility medications, and in vitro fertilisation [46]. Screening tests, based on the analysis of CA-125 (carbohydrate antigen 125) and transvaginal ultrasound, have not contributed to reducing ovarian and tubal cancer mortality rates [47]. Moreover, CA-125 lacks the necessary specificity and sensitivity to reliably detect ovarian cancer at an early stage [48]. Various markers have been analysed individually and in combinations with CA-125 to determine the optimal screening panel. These biomarkers include mesothelin, human epididymis protein 4 (HE4), osteopontin (OPN), or transthyretin (TTR) [49,50,51,52]. CA-125, HE4, transferrin, transthyretin, and β2-microglobulin have been incorporated into RMI (Risk of Malignancy Index), ROMA (Risk of Ovarian Malignancy Algorithm), or OVA1 algorithms used in the diagnosis of ovarian cancer [53]. The new potential immunological and molecular parameters that could be used in the diagnosis of OC, especially at the early stages, are still being searched for. Recently, researchers have been paying attention to parameters such as adipokines, galectins, CSCs, autoantibodies, miRNA, or ctDNA [54,55,56,57,58,59]. In the conducted study, the combination of HE4, CA-125, carcinoembryonic antigen (CEA), and vascular cell adhesion molecule 1 (VCAM-1) achieved a sensitivity of 93% and specificity of 98% [60]. The usefulness of a composite marker (CM) that combines CA-125 and soluble mesothelium-related (SMR) marker has been evaluated in the sera of ovarian cancer patients, and, at 98% SP, the CM identified 86,5% (45 of 52) of the cases [61]. The new biomarkers and their combinations that would improve the detection of ovarian cancer and reduce mortality in women are still being sought. Recently, liquid biopsy and ctDNA analysis have become increasingly common.

#### ctDNA and Ovarian Cancer

Zhu JW. et al. [11] assessed the presence of ctDNA and visible residual disease in patients with serous ovarian cancer (*n* = 48). The researchers analysed the DNA of 59 genes associated with breast and ovarian cancer. Tumour-specific variants (TSVs) were identified in the cancer cells of 47 patients. The analyses were performed on plasma samples collected from 47 women after surgery and before they started chemotherapy. The authors also compared survival rates among women without residual disease, with detectable ctDNA and with undetectable ctDNA. A total of 15 patients (31.9%) of the 47 had visible residual disease and detectable ctDNA. Of the remaining 31 patients (65.9%) with no visible residual disease, 24 (77.4%) patients had detectable ctDNA. Patients with no visible residual disease but detectable ctDNA had a higher mortality rate (16 of 24 died) than those without detectable ctDNA (three of seven died). According to the researchers, the presence of ctDNA in post-surgical serum samples may indicate the presence of microscopic residual disease and may predict recurrence. However, the authors emphasise that larger studies are needed to elucidate their findings [11]. Chao A et al. [62] conducted a study involving 29 patients with various histological types of OC (13 HGSC, nine CCC, two MC, four LGSC, and one ENOC) to determine whether plasma ctDNA mutations could offer prognostic insights for these patients. The researchers examined whether mutations detected in circulating tumour DNA (ctDNA) samples were concordant with those identified in the corresponding formalin-fixed, paraffin-embedded tumour specimens. At least one detectable ctDNA variant was found in 24 (82.8%) of the women in the study. Additionally, the proportion of mutations identified in pre-treatment ctDNA samples that were consistent with corresponding tumour specimens was 92.3% for HGSC patients and 58.6% for the entire cohort. Moreover, women with advanced-stage disease were more likely to have detectable ctDNA mutations before surgery and after surgery before adjuvant chemotherapy. Patients with HGSC were more likely to have ctDNA mutations detected before surgery. The researchers conclude that ctDNA mutations are common in patients with OC, and their presence after surgery was an independent predictor of less favourable progression-free survival (PFS) and overall survival (OS) [62]. A study on the use of ctDNA analysis in detecting residual disease after treatment in patients with epithelial ovarian cancer (EOC) was conducted by Kallio HM et al. [63], who analysed 264 plasma samples collected from 63 women. ctDNA analysis was performed on the samples collected at diagnosis (pre-surgery), at the first, third, and sixth cycles of chemotherapy, at progression and during follow-up at one-year intervals after the completion of first-line treatment. The researchers found that ctDNA was detected in 51 of 55 (93%) of the samples at diagnosis and in all 18 of the samples at progression. Moreover, the presence of ctDNA in the last on-treatment sample was associated with shorter time to progression and reduced OS in patients with HGSC. In 12 patients, the ctDNA assay detected progression of the disease earlier than standard surveillance methods (CA-125 test, gynaecological examinations, computed tomography scans, and transvaginal ultrasonography). The authors emphasise that ctDNA assays can achieve high SN and SP in the detection of residual disease after surgery for ovarian cancer. Compared with CT, ctDNA assays are cheaper and do not expose patients to ionising radiation [63]. Pereira E et al. [27] examined 44 women with gynaecologic cancers, including 22 patients with OC. The researchers detected circulating tumour ctDNA in 93.8% of patients, and its levels were found to be highly correlated with CA-125 serum levels and CT scan results. Moreover, they found that undetectable levels of ctDNA six months following initial treatment were associated with improved PFS (*p* = 0.0011) and OS (*p* = 0.0194) [27]. Ratajska M et al. [64] assessed ctDNA in patients with OC for *BRCA1/2* mutational analysis by NGS. The pathogenic *BRCA1/2* variants were detected in 30 of 121 (24.8%) patients. The study showed that identifying germline and somatic BRCA1/2 mutations in ctDNA from patients could be a valuable additional method of detecting somatic alterations. Moreover, ctDNA can potentially be used to monitor the efficacy of PARP1 inhibitors and detect secondary reversion of BRCA1/2 mutations [64]. The prognostic significance of ctDNA has also been described by Gifford G et al. [65], who analysed plasma DNA of patients with EOC for methylation of the hMLH1 CpG island before carboplatin/taxoid chemotherapy and at relapse. The researchers showed the association between the acquisition of *hMLH1* methylation after chemotherapy and survival after progression, concluding that the acquisition of hMLH1 methylation in plasma DNA at relapse predicts poor OS of patients, independent from time to progression and age (*p* = 0.007). The absence of visible residual disease after surgery indicates a relatively good prognosis. Determination of ctDNA can be a useful tool for predicting the presence of latent residual disease, risk stratification after treatment, and recurrence of disease [65]. The relationship between ctDNA and patient outcomes, as well as CA-125 status, both before surgery and during post-treatment surveillance, was investigated by Hou JY et al. [66] in order to determine its use as a prognostic biomarker for EOC. The authors found that both detection and higher ctDNA levels in pre-surgical plasma were associated with higher grade histology, stage, and increased likelihood of mortality. Additionally, the researchers found that the presence of circulating tumour ctDNA after surgery is highly associated with reduced recurrence-free survival, and that ctDNA is a better marker than CA-125 for identifying patients at the highest risk of recurrence. The authors suggest that monitoring ctDNA could be helpful when making clinical decisions for patients with EOC [66]. It is believed that ctDNA is a promising biomarker for diagnosing, monitoring response to treatment, and predicting prognosis in EOC [67]. The significance of ctDNA in ovarian cancer is summarised in Table 3 [11,27,62,63,64,65,67].

### 2.2. Endometrial Cancer (EC)

Endometrial cancer is a malignant tumour of the inner epithelial lining of the uterus, comprising different histological subtypes and molecular phenotypes. Historically, EC was categorised as Type I, consisting of grade I or grade II endometrioid adenocarcinomas, or Type II, including grade III endometrioid adenocarcinomas, serous clear cell, undifferentiated, and carcinosarcomas [68].

#### ctDNA and Endometrial Cancer

Moss LE et al. [69] investigated the usefulness of ctDNA for detecting and monitoring EC recurrence and progression in a group of 13 women. Targeted next-generation sequencing (tNGS) and personalised ctDNA assays were used. In the study, at least one somatic mutation at a variant allele frequency (VAF) > 20% was detected in 69% of patient tumours, while the other four patients were whole-exome sequenced and all of them harboured mutations in genes not analysed by tNGS. Moreover, the study revealed the potential of ctDNA analysis as a biomarker for early detection and monitoring of EC recurrence; however, more research is required [69]. Jamieson A et al. [70] investigated the clinical usefulness of detecting ctDNA mutations in EC and OC patients. The researchers found an association between the preoperative detection of ctDNA mutations, advanced stage and higher preoperative CA-125 levels, as well as subsequent disease recurrence. According to the authors, analysing plasma for ctDNA mutations may help to identify patients with more advanced-stage disease and enable real-time monitoring of treatment response and earlier identification of disease recurrence in both EC and OC [70]. Feng W et al. [28] assessed the feasibility of personalised ctDNA detection for monitoring recurrence and evaluating prognosis in high-risk EC. The researchers detected ctDNA in six of nine (67%) baseline plasma samples and in four of nine (44%) serial postoperative plasma samples, which was valuable in monitoring high-risk EC relapse during postoperative follow-up. Moreover, the study showed that ctDNA was a better biomarker than serum CA-125 and HE4, as postoperative ctDNA detection to estimate tumour relapse had 100% SN and 83.3% SP [28]. The pilot study was conducted by Grassi T et al. [71]. The study group consisted of 11 patients with suspected high-grade EC. ctDNA was detected in 6 of 10 (60%) pre-surgery patients and in 3 of 11 (27%) cases post-surgery. Moreover, the study showed that pre-surgery detection of ctDNA was consistent with advanced stage (80%), serous histology (80%), lymphatic spread of disease (100%), deep myometrial invasion (100%), and lympho-vascular space invasion (75%). According to the authors, the results of the study suggest an association between the detection of ctDNA prior to surgery and the presence of cancer with aggressive tumour characteristics or an advanced stage; however, further studies are needed [71]. Casas-Arozamena C et al. [72] analysed uterine aspirates from patients with EC (*n* = 60) that were taken during surgery and analysed by NGS, revealing the presence of genetic alterations in 93% of the tumours. Moreover, the researchers examined blood samples collected at surgery for ctDNA and CTC analysis. The study showed that ctDNA was present in 41.2% of cases, mainly in patients with high-risk tumours, which suggests an association with a more aggressive disease [72].

Despite the rising global incidence of EC and the poor OS of patients who experience a recurrence, no reliable biomarker has yet been identified that can effectively detect and monitor the progression of EC. Although reports indicate that ctDNA detection is a sensitive method for monitoring cancer activity and stratifying patients likely to respond to therapy, further research is needed. The significance of ctDNA in endometrial cancer is summarised in Table 3 [69,70,71,72].

### 2.3. Cervical Cancer (CC)

Human papillomavirus (HPV) is the causative factor of cervical cancer, which is one of the leading causes of gynaecological cancer deaths worldwide. Moreover, many genetic factors are involved in the development of this disease. Sensitive and specific biomarkers for CC are still being sought, with most studies being performed on. Blood is of research interest as a specimen for the detection of cancer biomarkers, including ctDNA detection by high-throughput genomic technologies [73].

#### ctDNA and Cervical Cancer

An interesting study was conducted by Jeannot E et al. [74] who analysed samples of serum of 94 HPV16- or HPV18-related CCs, which were collected after treatment and during an 18-month follow-up period. The authors assessed the relevance of the circulating HPV E7 gene as a marker of residual disease by ddPCR. HPV ctDNA was detected in 59 of 94 (63%) patients before treatment. The researchers found that detecting HPV ctDNA in serum samples was related to high FIGO stage (*p* = 0.02) and para-aortic lymph node involvement (*p* = 0.01). Moreover, there was a positive correlation was observed between the level of HPV ctDNA and the number of HPV copies in the tumour (r = 0.39, *p* < 0.001). Complete absence of HPV ctDNA at the end of treatment was significantly associated with a PFS (*p* < 0.0001). In addition, the study showed that patients with persistent HPV ctDNA in their serum experienced relapse at a median of 10 months after HPV ctDNA was detected. According to the authors, detecting HPV ctDNA is useful for predicting relapse in CC [74]. Similar results were obtained by Han K et al. [75], showing that the persistent HPV ctDNA after chemoradiation (CRT) is associated with inferior PFS in CC [75]. Similar reports were published by Cabel L et al. [76], who showed that in patients with locally advanced CC, HPV ctDNA level was related with HPV copy number in the tumour (r = 0.41, *p* < 0.001), and the detection of HPV ctDNA before chemoradiotherapy (CRT) was associated with tumour stage (*p* = 0.037) and lymph node status (*p* = 0.02). HPV ctDNA detection after CRT and during follow-up was related to lower DFS (*p* = 0.048) and OS (*p* = 0.0013). The authors state that residual HPV ctDNA detection at the end of CRT or during follow-up could help to identify patients who are more likely to experience a relapse [76]. Sivars et al. [77] investigated whether ctHPV DNA could be detected before, during, and after treatment in patients with CC or pre-malignant lesions. The study included 18 women with locally advanced CC (LACC), 15 with early stage CC (ESCC), and 21 with pre-malignant lesions. The researchers found ctHPV DNA in pre-treatment plasma of 94.4% of patients with LACC and 26.7% of patients with ESCC, while in patients with pre-malignant lesions, all samples were negative. Moreover, in pre-treatment plasma, patients with total ctDNA levels above the median had a worse PFS in comparison with patients whose total ctDNA levels were below the median. The researchers concluded that ctDNA is a promising prognostic biomarker in LACC, but further research is needed [77]. Bellone S et al. [78] investigated the use of personalised ctDNA in monitoring response to treatment, early recurrence, and survival in patients with uterine serous carcinoma (USC) and carcinosarcomas (CSs). The researchers showed that serial measurement of ctDNA may be a useful biomarker to monitor response to treatment and for the identification of early relapses in these diseases. Additionally, undetectable levels of ctDNA at the time of primary cancer treatment completion (including surgery, followed by platinum/taxane doublet chemotherapy) may be a novel predictor of survival differences in these patients. Moreover, has the potential to help distinguish between benign and malignant processes as identified through CT scan imaging during follow-up in some patients [78]. The significance of ctDNA in cervical cancer is summarised in Table 3 [74,75,76,77,78].

### 2.4. Breast Cancer (BC)

Breast cancer is a dynamic and highly heterogeneous disease that exhibits unique somatic alterations, including epigenetic modifications and gene expression changes, leading to recurrence and drug resistance. The methods used in BC diagnosis are mainly tissue biopsy and conventional imaging tools, which are not always able to provide sufficient information on changes in tumour biology over time [79]. Furthermore, these techniques do not assess the molecular heterogeneity of the tumour [6]. A breast cancer diagnosis relies on a radiological evaluation using mammography, ultrasound, or magnetic resonance imaging (MRI), which is then confirmed by a histopathological examination [80]. Molecular subtype of breast cancer is determined by the histological analysis of classical markers such as oestrogen and progesterone (ER and PR) receptors, human epidermal growth factor type 2 receptor (HER2), and proliferation index (Ki67) [81]. Serum biomarkers, such as carcinoma antigen 15-3 (CA15-3), carcinoma antigen 27-29 (CA27-29), and carcinoembryonic antigen (CEA), are useful in monitoring treatment response [81,82]. However, there is still a need to search for novel biomarkers for breast cancer diagnosis and monitoring of tumour progression and response to therapy [83]. Mutations in genes, such as *TP53*, *PTEN*, *BRCA1*, *BRCA2*, *NBS1*, *LKB1*, *ATM*, and others, are responsible for the majority of BC cases [26]. Detecting the disease at an early stage is very difficult, but it is crucial to reduce mortality rates depending on the type and stage of the disease (stage II and III breast cancers progress much faster than stage I cancers) [84]. Researchers are paying attention to the non-invasive liquid biopsy method, which may improve real-time disease monitoring, overcoming the drawbacks of invasive procedures. In comparison with traditional tumour biopsy, liquid biopsy presents some advantages to better overcome breast tumour heterogeneity, as markers from all tumour sites are released into the blood, and it is an easier to access and less painful procedure [6,85]. Potential liquid biopsy biomarkers in BC include cf-DNA, ct-DNA, CTCs, miRNA, lncRNAs, platelets, mRNA, proteins, and VOCs [6].

#### ctDNA and Breast Cancer

Sant M et al. [86], in a review on the role of ctDNA in BC, suggest its potential use for early diagnosis, the detection of minimal residual disease, the early diagnosis of relapse, and monitoring and treatment planning for advanced disease [86]. Chiu J et al. [87] conducted research in Asian patients with hormone receptor-positive, human epidermal growth receptor-2-negative (HR+/HER2−) advanced BC, evaluating the efficacy and safety of ribociclib and endocrine therapy (ET). The researchers concluded that ctDNA may be useful for monitoring tumour status and detecting alterations with treatment implications [87]. Coombes RC et al. [88] detected ctDNA in 16 of 17 BC patients with a lead time of up to 24 months prior to distant metastatic relapse, showing the ability of ctDNA to predict BC recurrence earlier than imaging, CA 15-3 levels, clinical examination, and liver function tests [88]. Zhou Y et al. [89] showed that ctDNA is a promising non-invasive alternative to evaluate the efficacy of neoadjuvant chemotherapy (NCT) in BC. The study was conducted on 63 tissue and 206 blood samples from 32 patients with locally advanced and metastatic BC collected at baseline (pre-treatment), during chemotherapy, after chemotherapy, after operation, and during follow-up. The researchers performed targeted deep sequencing with a custom 1021-gene panel. The results showed that the most common mutant genes in primary tumours were *TP53* (43.8%) and *PIK3CA* (40.6%). Moreover, patients with no tissue mutations during or after chemotherapy experienced a greater decrease in primary tumour size, while five patients who tested positive for mutations after chemotherapy developed distant metastases. The study revealed that sequential monitoring of blood ctDNA was an effective method of assessing NCT efficacy and relapses in patients [89]. In addition, a meta-analysis conducted by Papakonstantinou A et al. [17] showed that the detection of ctDNA, at baseline and after neoadjuvant therapy, was associated with worse RFS and OS. According to the authors, in patients with early BC, ctDNA assessment during neoadjuvant therapy may be helpful in stratifying the individual’s risk of relapse and tailoring patients’ treatment, but further prospective trials are needed [17]. Venetis K et al. [90] analysed the role of ctDNA for detecting ESR1 mutations and their implications for tailoring effective therapeutic strategies for HR+/HER2− metastatic BC, emphasising that ctDNA analysis is a minimally invasive method for the detection of ESR1 mutation [90]. Saura C. et al. [29] presented an interesting report on their studies of BC, which occurred during pregnancy (PrBC) or postpartum (PPBC). The researchers reported, for the first time, that ctDNA is present in breast milk (BM) from patients with BC. The study showed that analysing ctDNA from BM using ddPCR detects tumour variants in 87% of cases. Moreover, retrospective analysis of ctDNA in BM by NGS recapitulates tumour variants with an overall clinical SN of 71.4% and SP of 100%. According to the authors, BM may be a potential new source for liquid biopsy for PPBC detection [29]. Zhang X et al. [91] performed a study including 102 patients (861 serial plasma and matched tissue specimens) with early-stage BC who needed chemotherapy and 50 patients with benign breast tumours. The results demonstrated that ctDNA analysis is a sensitive and specific biomarker for diagnosis and differential diagnosis of early-stage BC patients who require chemotherapy [91]. Guan G et al. [92] analysed the usefulness of urinary ctDNA to monitor for residual disease. The study was conducted prospectively in 300 patients with early-stage BC. Measurements were taken prior to treatment and at various time points thereafter, for a total of eight measurements and the results were compared with healthy volunteers and patients whose urine specimens showed no detectable mutations. According to the authors, urinary ctDNA analysis potentially aids in complementing current monitoring of cancer relapse and may be helpful in early intervention [92]. Liu et al. [93] investigated the usefulness of urinary DNA for managing BC and predicting relapse. The authors also compared the sensitivity of plasma DNA with urinary DNA (*n* = 200 patients receiving neoadjuvant chemotherapy). The study found a strong correlation between plasma and urinary DNA at baseline, with the correlation coefficient r = 0.859. The researchers suggest that urinary DNA is potentially useful to measure the severity of early BC, and monitoring of early BC patients may help to predict disease relapse [93]. Cullinane C et al. [94] performed a systematic review and meta-analysis to investigate the association of ctDNA with DFS and PFS in early, locally advanced, and metastatic BC. The researchers concluded that elevated plasma ctDNA was correlated with a high risk of relapse, which suggests that plasma ctDNA may provide a method to stratify risk and personalise patient follow-up [94]. An interesting report was published by Siravegna G et al. [95], who suggest that the combined analysis of ctDNA in plasma and CSF may be helpful in the management of patients with HER2-positive metastatic BC. Moreover, liquid biopsies of CSF-derived circulating tumour DNA (ctDNA) contain clinically relevant genomic alterations in patients with breast cancer (BC) and central nervous system (CNS) metastases, and may be useful in the clinical management of brain metastases [95].

Patients whose cancer cells have a receptor for the hormones oestrogen and/or progesterone are usually treated with endocrine therapy (ET), which uses an aromatase inhibitor to reduce the body’s production of oestrogen and a cyclin-dependent kinase 4 and 6 (CDK4/6) inhibitor to block the growth of cancer cells [96]. Resistance to ET is associated with a higher risk of recurrence and increased mortality [97]. One of the main ways in which cancers can become resistant to ET is through mutations in the ESR1 gene, which encodes the oestrogen receptor. Researchers are interested in testing ESR1 mutations in liquid biopsies based on analysis of circulating cfDNA in plasma, which has been recommended to guide therapy in oestrogen receptor-positive breast cancer patients with ET-mediated progression [98]. Phase III trials are evaluating the efficacy and safety of new therapies or combinations of therapies for the treatment of breast cancer [96,99]. Analysis of ESR1 using liquid biopsy would allow regular testing to monitor ET sensitivity and guide treatment strategies [97].

Liquid biopsies in BC showed promising results, especially in monitoring the response to treatment and predicting progression or recurrence of the disease. It is believed that, with improvements in technologies for isolating tumour materials and further studies of this technique, liquid biopsies may play a bigger role in the BC clinic3. Conclusions

A summary of the significance of ctDNA in gynaecological cancers and breast cancer is presented in Table 3 [11,27,62,63,64,65,67,69,70,71,72,74,75,76,77,78,86,88,89,91,93].

**Table 3:** Summary of the importance of ctDNA in gynaecological and breast cancers.

Type of Cancer	Role of ctDNA	Ref.
**Ovarian cancer**	Prediction of the presence of microscopic residual disease and recurrence in a patient with OC	[11]
	Undetectable ctDNA 6 months after initial treatment is related to better PFS and OS	[27]
	Association of the presence of ctDNA mutations after surgery with worse PFS and OS	[62]
	The presence of ctDNA in the last sample during first-line treatment is associated with rapid progression and shortened OS in HGSC patients	[63]
	Monitoring of the efficacy of PARP1 inhibitors and detection of secondary reversion of BRCA1/2 mutations	[64]
	Association of the detection of hMLH1 methylation in plasma DNA at relapse with worse OS	[65]
	Diagnosis, monitoring response to treatment, and prognosis in EOC	[67]
**Endometrial cancer**	A potential biomarker for early detection and monitoring of EC relapse	[69]
	Real-time monitoring of treatment response and earlier identification of relapse in EC and OC	[70]
	Evaluation of tumour recurrence in EC (SN 100%, SP 83.3); monitoring of high-risk EC recurrence during postoperative follow-up	[28]
	Association between preoperative ctDNA detection and stage and presence of cancer with aggressive tumour characteristics	[71]
	Association with more aggressive disease	[72]
**Cervical cancer**	Presence of HPV ctDNA related to high FIGO stage; association of complete absence of HPV ctDNA before the end of treatment with longer PFS	[74]
	Detectable HPV ctDNA at the end of chemoradiotherapy is associated with worse PFS	[75]
	Presence of HPV ctDNA before chemotherapy is associated with tumour stage; presence of HPV ctDNA after completion of therapy and during the follow-up period is associated with shorter DFS and OS	[76]
	HPV ctDNA as a prognostic biomarker for locally advanced CC	[77]
	Monitoring of response to therapy; identification of early relapses	[78]
**Breast cancer**	BM may be a potential new source for liquid biopsy for PPBC detection	[29]
	Detection of minimal residual disease, early detection of recurrence, monitoring, and treatment planning of advanced disease	[86]
	Ability to predict relapse	[88]
	Evaluation of the effectiveness of NCT and relapse	[89]
	Diagnosis and differential diagnosis of early-stage BC patients	[91]
	Potential usefulness of urine DNA in early-stage breast cancer; prediction of disease recurrence	[93]
	ESR1 mutation in baseline ctDNA is associated with worse PFS and OS in patients treated with exemestane compared with fulvestrant	[100]

The implementation of LB in precision medicine has demonstrated significant potential for various types of cancer, including gynaecological and breast cancers.

In ovarian cancer, ctDNA has been used as a prognostic factor for PFS and OS, as a diagnostic factor, and also in the monitoring of therapy (Table 3). Finding ctDNA in body fluids before treatment may help to diagnose ovarian cancer early and choose the right treatment for the patient. However, more research is needed [59].

ctDNA in endometrial cancer acts as a prognostic factor and is useful for treatment monitoring, detection, and monitoring of recurrence (Table 3). Studies have shown an association of ctDNA after hysterectomy with poorer clinical outcomes [101].

The use of ctDNA in CC is found as a prognostic factor and also as a factor for monitoring response to therapy and identifying early relapses (Table 3). An additional advantage of the actual content of tumour biomarkers consisting of ctDNA from both tumour tissue and viral agent in HPV-positive tumours may be minimally invasive, rapid, and inexpensive sample collection [24].

The use of LB and ctDNA analysis in breast cancer ranges from early detection and monitoring of residual disease to treatment planning for advanced disease (Table 3).

Liquid biopsy seems to be applicable at every stage of the care of a patient. Screening patients is used to predict disease risk, prognosis, and OS in early disease to inform targeted therapies in the first-line setting, identify new biomarkers, and monitor response to treatment. During disease progression, in advanced cancer, liquid biopsy is used to confirm the diagnosis of metastases, inform targeted therapy selection, monitor response and resistance to treatment, and after treatment is completed, it can be used to identify minimal residual disease, monitor patients in remission, and identify the risk of relapse [102]. As a novel and minimally invasive method for the detection of tumour biomarkers in peripheral blood, it has been demonstrated to enable early detection and tumour profiling, as well as the selection and monitoring of therapy. The limitations of the method include low amounts of CTCs, ctRNA, and ctDNA, a lack of diverse standardisation and isolation procedures, and elevated economic costs.

As the abundance of ctDNA is very low, the use of highly sensitive techniques for the detection of tumour mutations is crucial. The determination of ctDNA has been shown to set new standards for diagnosis and treatment in patients diagnosed with gynaecological and breast cancers, thanks to innovative sequencing techniques.

The economic factor also appears to be an important consideration. Many researchers point to lower costs of sample isolation compared to tissue biopsy [7]. However, some authors point to the high costs of performing molecular tests, which prevent the introduction of this method into clinical practice. According to Sánchez-Calderón D et al. [103], the inclusion of liquid biopsy in the treatment of HER2-positive advanced breast cancer in Colombia was not cost-effective. The authors emphasise the need for studies to provide more evidence on the utility of this test, depending on the financial capacity of Colombia or other countries [103]. However, due to its limitations, this method is not yet considered a standard tool for confirming and diagnosing diseases, including cancer, and is currently used as a complementary test to tissue biopsy [5].

The studies cited in this review included a wide range of patient numbers and were conducted in small groups using various methods and settings. Further studies involving larger patient numbers and covering longer time periods are required to establish the utility of liquid biopsy in routine gynaecological and breast cancer practice.

The integration of this assay into clinical practice in modern oncology presents challenges, yet it offers numerous benefits, including the mitigation of risks associated with invasive procedures, enhanced diagnostic and therapeutic efficacy, and an improvement in the quality of life for oncology patients. Further research is necessary to fully explore these benefits.

## Figures and Tables

**Table 1:** Characteristics of cfDNA and ctDNA molecules.

Characteristics	cfDNA	ctDNA	Refs.
Molecular structure	Single- and double-stranded DNA that circulates freely in the bloodstream, released from various cells in the body as a result of apoptosis and necrosis [14]	Fragments of single- or double-stranded DNA released into the bloodstream by tumour cells [14]; single- or double-stranded DNA found in plasma or serum [15]	[14,15]
Molecular size	About 150–200 bp [14] and 150–180 bp [10]	Shorter than cfDNA [15]; about 146 bp [14]; and 160 to 200 base pairs [11]	[10,11,14,15]
Serum level	Healthy subjects: 0–100 ng/mL; cancer patients: 0–5 ng/mL to more than 1000 ng/mL [13]	0.01% of the total cfDNA detected in the patient’s plasma [14]	[13,14]
Half-life	5–150 min [14]	Approximately 15 min to 2.5 h [10]; 23–52 min after surgical resection of the tumour [14]; and 16 min to 2.5 h [16]	[10,14,16]

**Table 2:** Comparison of the sensitivity and specificity of ctDNA testing in gynaecological and breast cancers.

Type of Cancer	Study Group	Method	SN/SP (%)	Ref.
Gynaecological cancers/ovarian cancer	44 (including 22 with ovarian cancer)	ddPCR	91/60	[27]
High-risk endometrial cancer (assessment of postoperative tumour recurrence)	9	ddPCR	100/83.3	[28]
Breast cancer Breast milk	19 (10 diagnosed during pregnancy and 9 diagnosed during breastfeeding)	NGS	71.4/100	[29]
Cervical cancer	684 (fifteen studies; meta-analysis)	-	27/94	[30]

## Data Availability

No new data were created or analyzed in this study.
